# Dissecting the Permeability of the *Escherichia coli* Cell Envelope to a Small Molecule Using Tailored Intensiometric Fluorescent Protein Sensors

**DOI:** 10.1021/acsomega.3c05405

**Published:** 2023-10-11

**Authors:** Philipp Kemp, Wadim Weber, Charlotte Desczyk, Marwan Kaufmann, Josefine Panthel, Theresa Wörmann, Viktor Stein

**Affiliations:** † Department of Biology, TU Darmstadt, 64287 Darmstadt, Germany; ‡ Centre for Synthetic Biology, TU Darmstadt, 64283 Darmstadt, Germany

## Abstract

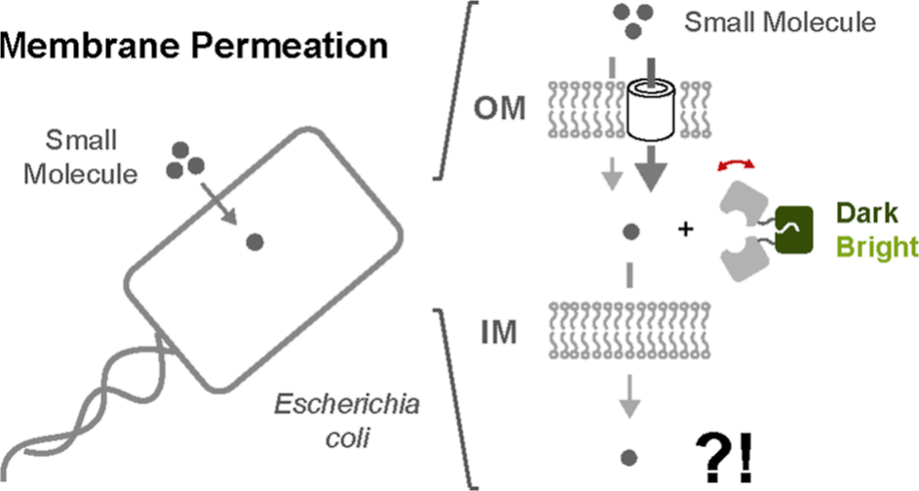

Membranes provide a highly selective barrier that defines the boundaries of any cell while providing an interface for communication and nutrient uptake. However, despite their central physiological role, our capacity to study or even engineer the permeation of distinct solutes across biological membranes remains rudimentary. This especially applies to Gram-negative bacteria, where the outer and inner membrane impose two permeation barriers. Addressing this analytical challenge, we exemplify how the permeability of the *Escherichia coli* cell envelope can be dissected using a small-molecule-responsive fluorescent protein sensor. The approach is exemplified for the biotechnologically relevant macrolide rapamycin, for which we first construct an intensiometric rapamycin detector (iRapTor) while comprehensively probing key design principles in the iRapTor scaffold. Specifically, this includes the scope of minimal copolymeric linkers as a function of topology and the concomitant need for gate post residues. In a subsequent step, we apply iRapTors to assess the permeability of the *E. coli* cell envelope to rapamycin. Despite its lipophilic character, rapamycin does not readily diffuse across the *E. coli* envelope but can be enhanced by recombinantly expressing a nanopore in the outer membrane. Our study thus provides a blueprint for studying and actuating the permeation of small molecules across the prokaryotic cell envelope.

## Introduction

Membranes constitute an exquisitely selective barrier that delineates a cell from the extracellular environment and provides an interface for communication and nutrient acquisition. Depending on their physicochemical properties, small molecules can permeate membranes either passively or require distinct membrane proteins. These range from comparatively nonspecific nanopores, e.g., porins in the outer membrane of Gram-negative bacteria^1^ to highly selective transporters that either facilitate diffusion or even mediate an active transport.^2^ Despite their central physiological role and many important implications for basic research and biotechnology, our ability to study the permeation of distinct small molecules across biological membranes remains rudimentary and underdeveloped to date.

**Figure 1:**
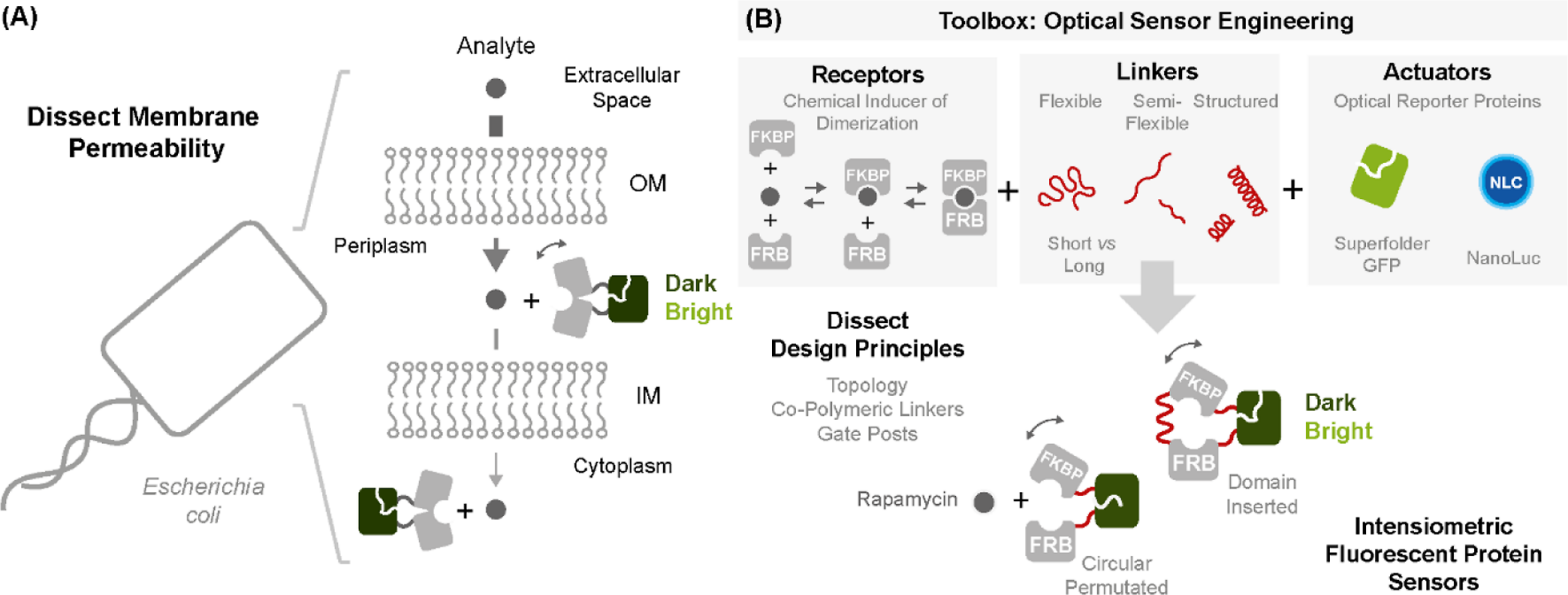
Framework for studying the permeation of a small molecule across the *E. coli* cell envelope. (A) Permeation of the *E. coli* cell envelope to a small molecule is probed using iFP sensors which can be flexibly targeted either to the cytoplasm or the periplasm; (B) a highly modular approach was pursued to construct intensiometric rapamycin detectors (iRapTors) through recombination of the structurally distinct rapamycin-specific FKBP12 and FRB receptors with sfGFP using copolymeric linkers of variable lengths and composition while systematically probing the role of domain-inserted and circular-permutated topologies.

Addressing these limitations, we devise a scalable framework to probe the permeability of the *Escherichia coli* cell envelope using intensiometric fluorescent protein (iFP) sensors (Figure 1A). The approach is exemplified for rapamycin which constitutes a biotechnologically important macrolide.^3^ It also underlies many research tools. Most prominently, it is used as a chemical inducer of dimerization (CID) which is capable of bringing any two proteins that have been tagged with the corresponding FKBP12 and FRB receptors into close proximity.^4^

To study the permeation of rapamycin across the *E. coli* cell envelope, we thus first develop a set of iFP sensors that can be flexibly targeted either to the cytoplasm or the periplasm (Figure 1B). As part of our construction efforts, we comprehensively dissect the design principles of iFP sensors focusing on the scope of minimal copolymeric linkers while dissecting the constraints imposed by topology and the need for gate post residues. The resultant iFP sensors—generically termed intensiometric rapamycin detectors (iRapTor)—are then used to probe the permeation of rapamycin across the inner and outer membrane of *E. coli*. Despite its lipophilic character, rapamycin does not readily permeate the cell envelope as both the inner and outer membranes pose a considerable diffusion barrier, which can however be alleviated through the recombinant expression of a protein nanopore in the outer membrane. Overall, our study provides a scalable blueprint for assessing and augmenting the permeation of a distinct small molecule across the *E. coli* inner and the outer membrane using iFP sensors.

## Results and Discussion

The macrolide rapamycin has gained prominence as an immunosuppressive agent^3^ and constitutes a widely used tool in both basic research and biotechnology. Most prominently, it is used as a CID and model receptor^4^ in the construction of many different types of protein switches and sensors with a range of applications in vitro, in mammalian cells,^4^ and in a few limited instances in *E. coli*. For instance in *E. coli*, rapamycin formed part of a rapamycin-inducible protein degradation system^5^ and could also be detected using transcriptional and post-translation sensors.^6,7^ In part, the widespread use of rapamycin as a model small molecule and tool in basic research can be attributed to its highly modular and well-defined FKBP12 and FRB receptors. Furthermore, its ease of administration implies facile diffusion across cellular membranes, at least in mammalian cells.

However, as we aimed to establish a set of rapamycin-responsive protease switches^8,9^ in *E. coli*, circumstantial observations suggested that rapamycin did not readily accumulate in the cytoplasm as its permeation across the outer, inner, or even both membranes appeared to be limited. The underlying molecular reasons however remain ambiguous and unclear. On the one hand, rapamycin with a size of 914 Da exceeds the diffusion cutoff of outer membrane porins at >600 Da;^1^ on the other hand, rapamycin is considered lipophilic^10^ which should in turn facilitate passive diffusion across lipid bilayer membranes.

**Figure 2:**
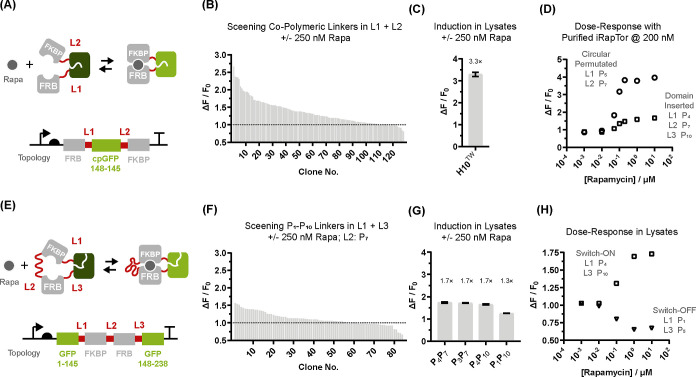
Engineering intensiometric rapamycin detectors (iRapTor): (A) design of circular-permutated iRapTor variants: the rapamycin-specific receptors FRB and FKBP12 were recombined with a circularly permutated version of superfolder GFP (sfGFP). Recombination was achieved using a combinatorial library of copolymeric linkers comprising flexible Gly-, semiflexible PAS-, and rigid poly-Pro motifs in L1 and L2 with a theoretical diversity of 400 linker variants (see Table S1); (B) summary of the combinatorial linker screen displaying the induction ratios of individual iRapTor variants ±250 nM rapamycin; (C,D) the best-performing iRapTor variant, termed H10TW, featured characteristic P_5_ and P_7_ linkers in position L1 and L2 and could be induced approximately 3.3-fold in cell lysates and close to four-fold in the purified form; (E) design of domain-inserted iRapTor variants: a single-chain FKBP12-P_7_-FRB receptor was inserted between position 145 and 148 of sfGFP. Insertion was optimized through a combinatorial library of P_1_ to P_10_ linkers in L1 and L3 while FKBP12 and FRB were separated by a fixed P_7_-linker in L2; (F) screening a saturating amount of poly-Pro linker combinations in the context of a domain-inserted topology displayed a large plasticity in the underlying response comprising both switch-ON and switch-OFF variants; (G) functional characterization of the best-performing domain-inserted iRapTor confirmed up to >1.7-fold induction for different switch-ON variants; and (H) quantitative dose–response curves for both switch-ON and switch-OFF variants. Error bars arise from two technical replicates.

To follow up on this further and gain a better understanding of the underlying constraints, we engineered a set of FP sensors in order to study the permeation of rapamycin across distinct membrane compartments of the cell envelope of *E. coli*. Given the limited number of FP sensors that demonstrably function both in the cytoplasm and the periplasm, we decided to focus on iFP sensors which are composed of a single fluorescent protein and polypeptide chain.^11^ To this end, FKBP12 and FRB were fused to the N- and C-termini of circular-permutated superfolder GFP (sfGFP) which previously proved functional in both the cytoplasm and periplasm^12^ (Figure 2A). Recombination was achieved using iFLinkC exploiting defined sets of flexible Gly-rich, semiflexible PAS-ylation, and rigid Pro-rich linkers (Table S1).^8,13^ A subsaturating number of 128 linker mutants was then screened in cell lysates for the maximum induction of fluorescence in the presence and absence of 0.25 μM rapamycin (Figure 2B, Table S1). Strikingly, the library displayed substantial plasticity, where induction ratios solely depended on the identity of the linker with the most potent iRapTor variant, termed H10TW, featuring a unique P_5_ and P_7_ linker in L1 and L2, respectively. Notably, iRapTor^H10TW^ could be induced 3.3-fold in cell lysates (Figure 2C) and nearly four-fold after purification (Figure 2D).

Motivated by the potency of poly-Pro linkers in the context of a circular-permutated topology, we then explored to what extent poly-Pro linkers could yield functional iFP sensors in the context of a domain-inserted topology (Figure 2E). To this end, a single-chain FKBP12-P_7_–FRB allosteric receptor module^8^ was flanked by combinations of P_1_ to P_10_ linkers and inserted between position 145 and 148 of sfGFP (Figure 2E). Again, screening a saturating amount of 420 iRapTor linker variants (relative to a theoretical diversity of 100) highlighted substantial plasticity in the response of individual iRapTor variants comprising both switch-ON and, strikingly, switch-OFF variants solely depending on the length of the poly-Pro linkers in L1 and L3 (Figure 2F). Notably, gate post residues at positions 145 and 148 turned out critical in the context of minimal poly-Pro linkers as their omission yielded an unresponsive iRapTor library (Figure S1). Sequencing a select number of domain-inserted iRapTor variants further revealed that the most functional domain-inserted iRapTor variants featured linkers with characteristic increments of three Pro residues (Tables S2 and S3). The latter is consistent with a left-handed poly-Pro II helix and implies a need for a precise relative orientation of the FKBP12 and FRB receptor domains. The response of individual iRapTor variants was subsequently confirmed in cell lysates and after purification. Here, induction ratios reached approximately 1.7-fold switch-ON and 0.66-fold switch-OFF while rapamycin dose–response curves generally turned out quantitative (Figure 2D,G,H). However, no further conclusions were possible regarding the strength of the underlying interaction between rapamycin and different iRapTor variants given a comparatively strong inflection of the underlying dose–response curve at the sensor concentration indicated a titration regime^14^ (Figure 2D).

**Figure 3:**
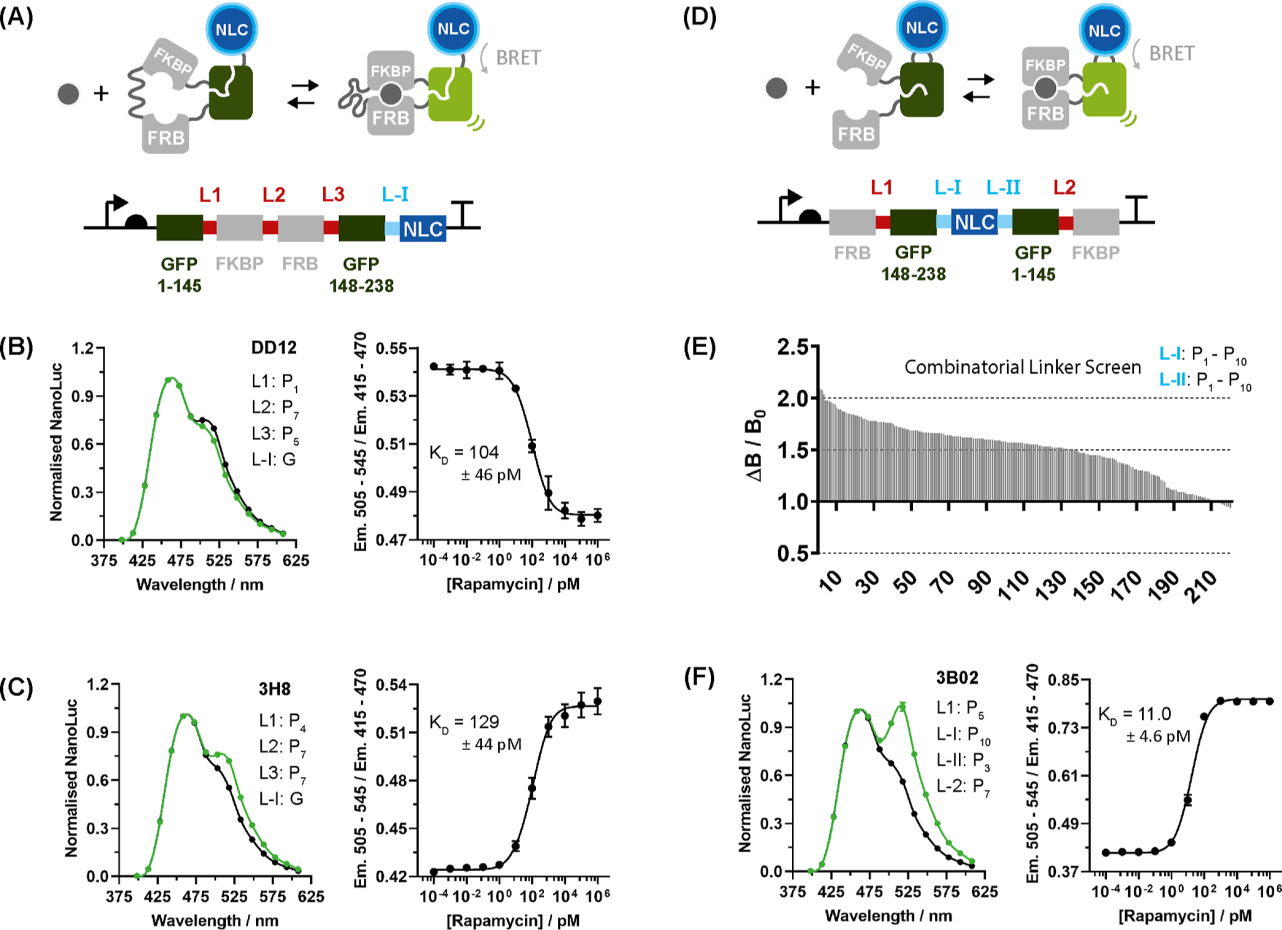
Characterizing the response of LuciRapTor. (A) Domain-inserted iRapTor variants were turned into bioluminescent sensors by appending NanoLuc to their C-terminus; (B,C) emission spectra and *K*_D_ curves of domain-inserted LuciRapTor switch-ON and switch-OFF variants with linker compositions indicated. The response was resolved using 10 pM LuciRapTor sensor while emission curves were recorded using ±2 μM rapamycin; (D) circular-permutated iRapTor^H10TW^ were turned into bioluminescent sensors following recombination of NanoLuc with the native N- and C-termini of GFP. Recombination of iRapTor^H10TW^ was optimized by combinatorial linker screening using a library of poly-Pro linkers featuring combinations of P_1_–P_10_ in L-I and L-II; (E) the combinatorial linker library was screened ±2 μM rapamycin and assessed for maximum induction. The distribution of induction ratios across the library is shown; and (F) emission spectra and dissociation curves of the circular permutated LuciRapTor^H10TW^ variant 3B02 with linker compositions indicated. The response was resolved with 10 pM purified LuciRapTor^H10TW^ sensor. Emission curves are shown using ±2 μM rapamycin.

Thus, to gain a more quantitative understanding how poly-Pro linkers shape the response of individual iRapTor variants, a select number of iFP sensors were recombined with NanoLuc to enhance the sensitivity of the optical read-out^15^ (Figure 3A–C). For domain-inserted variants, NanoLuc could be appended at the C-terminus. For circular-permutated variants, recombination with NanoLuc was achieved through a limited linker screen based on combinations of P_1_ to P_10_ linkers (Figure 3D–F). Strikingly, the resultant bioluminescent sensors—generically referred to as LuciRapTors—displayed >10,000-fold enhanced sensitivity which enabled us to determine the apparent *K*_D_s for rapamycin using pM LuciRapTor sensors. In the case of domain-inserted LuciRapTors, both switch-ON and switch-OFF bound rapamycin with a *K*_D_ of 100 and 130 pM (Figure 3B,C). This means, affinity was increased by approximately 2 orders of magnitude following intramolecular tethering of FKBP12 and FRB relative to the intermolecular complex (with a *K*_D_ of 12 nM).^16^ Otherwise, no significant difference was observed in affinity between switch-ON and switch-OFF variants, which suggests that contraction of the FKBP12-P_7_-FRB clamp is comparatively insensitive to the sign of responsiveness. In contrast, a circular-permutated LuciRapTor bound rapamycin with an apparent *K*_D_ of 11 pM which significantly exceeded the strength of the primary interaction between rapamycin and FKBP12 with a *K*_D_ of 200 pM^16^ (Figure 3F). Furthermore, a quantitative analysis highlighted how topology and specifically a Pro_7_-linker between FKBP12 and FRB can modulate the response by 1 order of magnitude when comparing a *K*_D_ of 11 pM for circular-permutated with a *K*_D_ of 100–130 pM for domain-inserted LuciRapTors.

**Figure 4:**
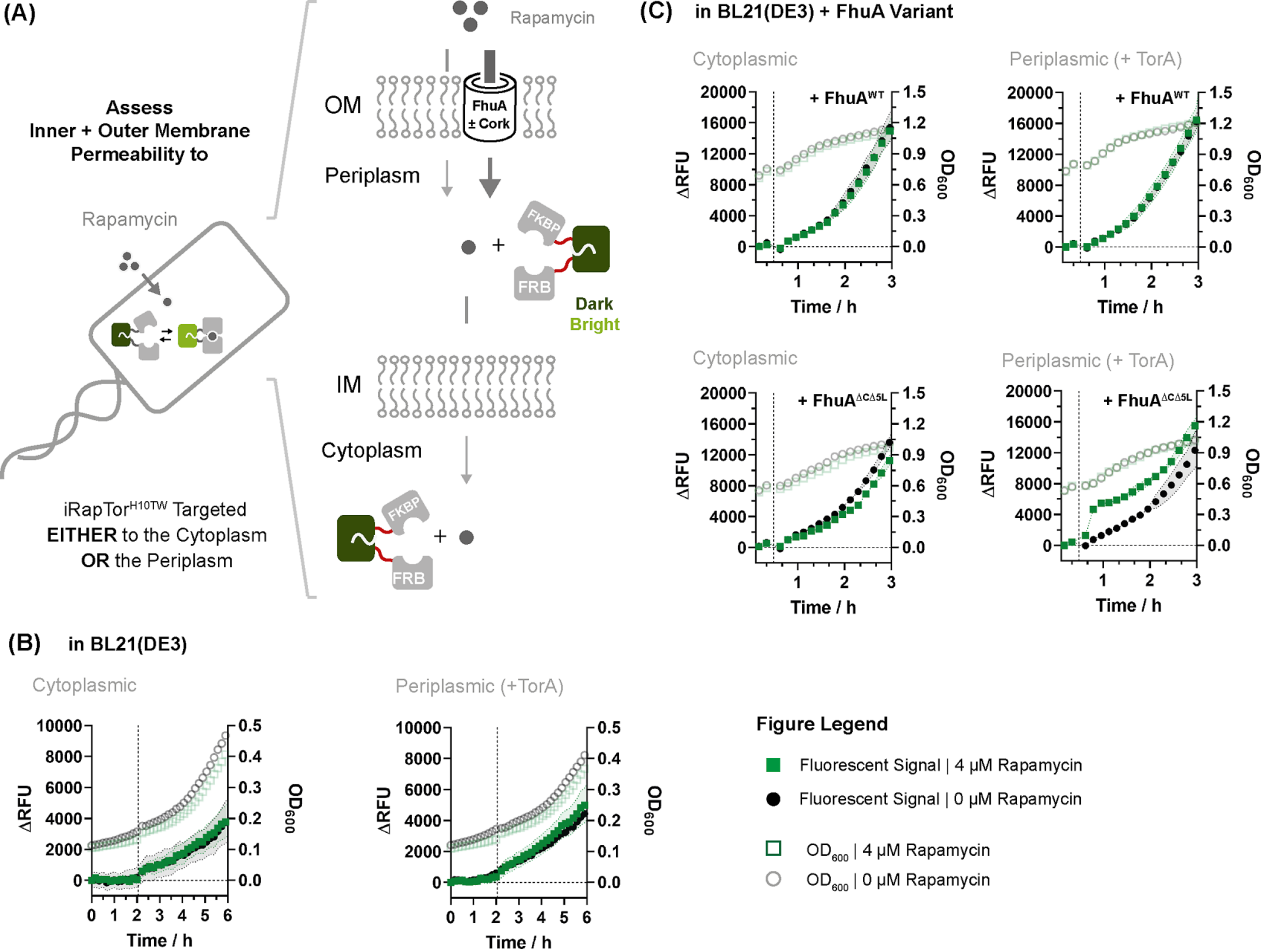
Evaluating the permeation of rapamycin across the *E. coli* cell envelope. (A) Permeation of rapamycin across the inner and outer membrane was probed with cytosolic and periplasmic iRapTor^H10TW^ in BL21(DE3). Periplasmic targeting was achieved using a TorA-based periplasmic export tag; (B) no increase in fluorescence was observed upon addition of 4 μM rapamycin either for cytosolic or periplasmic iRapTor^H10TW^; (C) to assess the effect of outer membrane permeabilization, iRapTor^H10TW^ was coexpressed either with wild-type FhuA^WT^ or a constitutively open cork-less FhuA^ΔCΔ5L^ variant. Only periplasmic iRapTor^H10TW^ in combination with a constitutively open cork-less FhuA^ΔCΔ5L^ triggers a rapamycin-dependent increase in fluorescence. Data show replicates of three independently picked colonies. Fluorescent signals are shown as full squares and full circles; OD_600_ is shown as empty squares and circles; dotted line indicates time point at which rapamycin was added; green traces denote samples treated with 4 μM rapamycin dissolved in DMSO; and black traces denote equivalent amount of 0.5% DMSO only.

Finally, we applied our newly developed iFP sensors to quantify the permeation of rapamycin across the inner and outer membranes of *E. coli* (Figure 4A). To this end, the best-performing sensor iRapTor^H10TW^ was expressed under a propionate-inducible promoter with and without a TorA-periplasmic export tag in BL21(DE3). Yet, no increase in fluorescence was initially detected following addition of up to 4 μM rapamycin, suggesting that rapamycin permeates neither the inner nor the outer membrane of BL21(DE3) in sufficiently large quantities to trigger a fluorescent signal (Figure 4B). To dissect this further and examine to what extent diffusion of rapamycin across the outer membrane may be facilitated by a nanopore, two different variants of the outer membrane protein FhuA^17^ comprising the closed wild-type FhuA^WT^ and a cork-less, constitutively open FhuA^ΔCΔ5L^ variant^18^ were coexpressed in *E. coli*. The latter was previously shown to increase susceptibility to antibiotics^19^ so we hypothesized that it could equally facilitate the diffusion of rapamycin across the outer membrane (Figure 4C,D). Strikingly, cork-less FhuA^ΔCΔ5L^ triggered a rapid increase in fluorescence following addition of rapamycin for periplasmic but not cytosolic iRapTor^H10TW^. Crucially, no increase in fluorescence was observed for FhuA^WT^, demonstrating that rapamycin diffuses across cork-less FhuA^ΔCΔ5L^. Furthermore, this means, rapamycin is not able to diffuse in sufficient quantities across the inner membrane of *E. coli* even when the outer membrane is partially permeabilized with FhuA^ΔCΔ5L^, highlighting its limited ability to diffuse across the lipid bilayer membranes of the *E. coli* cell envelope.

In concluding considerations, a scalable framework was devised to study and engineer the permeation of a small molecule across the *E. coli* cell envelope using iFP sensors. Proof-of-concept was achieved for the biotechnologically relevant analyte rapamycin. To this end, tailored iFP sensors were engineered by recombining FKBP12 and FRB with sfGFP using copolymeric linkers of variable lengths and amino acid composition. Notably, minimal poly-Pro linkers turned out highly functional in the context of different iFP sensors necessitating only limited screening efforts which further corroborate the potency of poly-Pro linkers in the construction of synthetic protein switches and sensors.^8,20,21^ In addition, the scope of topology along with the need for gate post residues was systematically dissected in the context of minimal poly-Pro linkers. Notably, topology exerts a significant impact on sensitivity with a >10-fold difference in the apparent *K*_D_ of the underlying iFP sensors. This can be attributed to a P_7_-linker separating FKBP12 and FRB in the domain-inserted topology as opposed to no linker in a circular-permutated topology. With a steady increase of CID modules that are being developed and integrated across different types of biosensors,^22^ this study exemplifies the facilitated construction of iFP sensors based on modular CID receptors in conjunction with minimal, copolymeric linkers. Notably, only limited screening is required to engineer well-performing iFP sensors^11^ with induction ratios > 4 that are comparable in the performance to many previously developed iFP sensors and turn out functional in live cell *E. coli* measurements.

The best-performing iRapTor variant, termed H10TW, was then used to assess the permeability of the *E. coli* cell envelope to rapamycin. Despite its lipophilic character, rapamycin did not readily permeate the outer membrane of *E. coli* in sufficient quantities in order to trigger a fluorescent signal but strictly required the expression of a large constitutively open nanopore variant FhuA^ΔCΔ5L^ in the outer membrane. Our study thus calls for caution in the development and application of rapamycin-based tools in *E. coli* which may ultimately turn out nontrivial due to its limited ability to permeate across the cell envelope of *E. coli*. In this regard, several factors need to be born in mind when assessing past applications of rapamycin in *E. coli*, for instance, in the context of both transcriptional and post-translational sensors^6,7^ and a rapamycin-inducible protein degradation system.^5^ First of all, one difference concerns the sensitivity of the underlying read-out. In case of a split T7 RNA polymerase system,^7^ a rapamycin-dependent response is first amplified through a transcriptional response and further facilitated using a highly sensitive NanoLuc reporter. Conversely, a cytosolic split-GFP reporter did not generate any measurable read-out. Similarly, in case of the rapamycin-inducible protein degradation system, miniscule amounts of rapamycin could suffice to trigger the knock down of endogenous proteins and affect a measurable phenotype.^5^ Further differences may arise from genetic modifications of the underlying strain as the implementation of a rapamycin-inducible degradation system in W3110-necessitated knockout of the ClpXP protease specificity-enhancing factor *ssp*B^5^ which may pleiotropically impact cell physiology in general and membrane permeability in particular.

## Conclusions

Overall, our study demonstrates how iFP sensors afford new experimental approaches to study and eventually engineer the permeation of distinct small molecules across microbial membranes. Pending the availability of suitable CID receptors and thereof based iFP sensors, we thus anticipate that our experimental framework will inspire new applications and help resolve key fundamental questions in both basic research and biotechnology. Notably, the molecular and genetic factors that underlie the permeation of distinct small molecules largely remain unknown while the import as well as export across the cell envelope has been implicated to be a rate-limiting step in microbial biotechnology.^23^ In further complementary applications, our study provides a route to study and engineer the functional properties of large nanopores located in the outer membrane of *E. coli* extending the FuN screen principle beyond the inner membrane.^24^ Notably, this includes β-barrel-based nanopores such as FhuA which has proven a highly versatile scaffold across a number of nanopore engineering endeavors with a range of applications in biosensing and biocatalysis.^25^

## Experimental Section

### Recombinant DNA Work

iRapTors, LuciRapTors, protein nanopores, and polycistronic transcripts were assembled by means of iFLinkC.^8,13^ A detailed list of protein coding sequences including untranslated regions that were used to connect individual transcriptional units in a polycistronic expression construct is provided in the Supporting Information. For the purpose of screening, iRapTors were expressed from a propionate-inducible promoter in the context of pProFL which refers to an iFLinkC compatible version of pPro24.^26^ To assess the permeation of rapamycin across the inner and outer membrane, iRapTor^H10TW^ was expressed from a propionate-inducible promoter in the context of pProFL while the two different FhuA variants, FhuA^WT^ and cork-less FhuA^ΔCΔ5L^, were coexpressed from a constitutively active promoter in the context of pConC which has been derived of the pCtrl2 backbone.^24^

### Screening iRapTor and LuciRapTor Linker Libraries

Combinatorial linker libraries were expressed from a propionate-inducible promoter based on pProFL. Briefly, libraries were transformed into BL21(DE3) and plated on LB agar plates supplemented with 100 μg/mL ampicillin (AMP) and incubated overnight at 37 °C. Single colonies were then used to inoculate 300 μL of LB (+100 μg/mL AMP + 50 mM sodium propionate) dispensed in 96 deep-well plates and grown overnight to saturation at 37 °C and 1050 rpm. The following day, cells were harvested by centrifugation at 4000 rpm, and the cell pellet resuspended in 200 μL of phosphate buffered saline (PBS) supplemented with 1 mg/mL lysozyme and 1 μg/mL DNase. Cells were lysed over the course of a 60 min incubation at 37 °C and shaken at 1050 rpm. The resultant cell lysates were then cleared of cellular debris by centrifugation at 15,000 *g* before 50 μL of the supernatant was duplicated across a black 96-well microtiter plate and mixed with 150 μL of PBS. The induction ratio of individual iRapTor variants was then quantified by fluorescence spectroscopy (excitation at 480 ± 10 nm/emission at 525 ± 10 nm) in the presence and absence of 250 nM rapamycin.

Bioluminescent LuciRapTors were screened analogously to fluorescent iRapTors with minor modifications. This means, after clearing cellular debris by centrifugation at 15,000 g, cell lysates were diluted 100-fold in PBS. Then, 5 μL of the diluted cell lysate was duplicated in a 384 microtiter plate before being mixed with 15 μL of PBS in the presence and absence of 2 μM rapamycin. Furimazine was generally used at a 2000-fold dilution of the commercial stock (Promega). The induction ratio of individual LuciRapTor variants was then quantified via the normalized emission ratio which is calculated as the bioluminescent signal at 505–545 nm (em. peak sfGFP) over the bioluminescent signal at 415–470 nm (em. peak NanoLuc) in the presence and absence of 2 μM rapamycin.

### Recombinant Expression and Purification of iRapTor Variants

To quantify the rapamycin-dependent response, a select number of iRapTor and thereof derived bioluminescent sensor variants were recombinantly expressed in *E. coli* and purified by His-tag affinity chromatography. Briefly, a single colony of BL21(DE3) transformed with a respective expression construct was used to inoculate a preculture of 5 mL of LB (+100 μg/mL AMP) and grown to saturation overnight at 37 °C and 180 rpm. The preculture was then used to inoculate 250 mL of LB medium (+100 μg/mL AMP) before being incubated at 37 °C and 180 rpm. When the OD_600_ reached a value of 0.4–0.6, the expression of individual iRapTor and LuciRapTor variants was induced with 50 mM sodium propionate and left to express overnight at 30 °C and shaking at 180 rpm. The following day, cells were harvested by centrifugation at 4000 *g* and stored at −20 °C until further use. For purification, cell pellets were washed twice in PBS and then resuspended in 50 mL of His-tag wash buffer (50 mM NH_2_PO_4_, 300 mM NaCl, 10 mM imidazole, pH 8.0) and lysed using an emulsiflex (Avestin). Lysates were then cleared of cellular debris by centrifugation for 15 min at 25,000 *g* and 4 °C. Individual iRapTor and LuciRapTor variants were then purified using an AKTA Pure L system according to manufacturer’s instructions (GE Healthcare). To this end, the supernatant was first passed over a Protino Ni-NTA 5 mL FPLC column (Machery & Nagel), washed using His-tag wash buffer, and then eluted using an imidazole gradient from 20 to 250 mM imidazole. Proteins were then transferred into storage buffer (100 mM Tris-HCl, 150 mM NaCl, 1 mM EDTA, 10% glycerol, pH 8.0) by gel filtration using PD-10 columns according to manufacturer’s instructions before being flash frozen in liquid nitrogen and stored at −80 °C.

### Functional Characterization of Fluorescent and Bioluminescent Rapamycin Sensors

The response of individual iRapTor and LuciRapTor variants was quantified in the purified form by means of fluorescent and bioluminescent spectrophotometry. Fluorescent iRapTor variants were assayed at 100 nM in a total volume of 200 μL of PBS, and the fluorescent signals were recorded at different rapamycin concentrations indicated. Bioluminescent LuciRapTor sensors were assayed at 10 pM in a total volume of 20 μL in luciferase assay buffer [50 mM Tris-HCl, 100 mM NaCl, 10% glycerol (v/v), 0.05% Tween 20, pH 7.4] at the rapamycin concentration indicated. Furimazine was used at a 2000-working dilution relative to the commercial stock (Promega). The functional state of LuciRapTor was quantified via the normalized emission ratio, which is calculated as the bioluminescent signal at 505–545 nm (em. peak sfGFP) over the bioluminescent signal at 415–470 nm (em. peak NanoLuc). The apparent *K*_D_ was determined through a nonlinear regression fit of the bioluminescent emission ratios to eq 1.
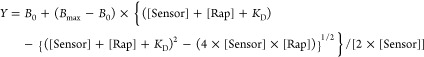
1

### Probing Membrane Permeability with iRapTor^H10TW^

iRapTor^H10TW^ was used to probe the permeability of the *E. coli* cell envelope to rapamycin. To this end, iRapTor^H10TW^ was expressed from a propionate-inducible promoter in the context of the pProFL backbone, while the wild-type FhuA^WT^ and a cork-less, constitutively open FhuA^ΔCΔ5L^ were coexpressed from pConC. To this end, constructs were transformed into BL21(DE3) as indicated and plated overnight on LB agar plates [+5 μg/mL AMP and 1.25 μg/mL chloramphenicol (CHL)]. The following day, single colonies were used to inoculate 300 μL of LB (+100 μg/mL AMP; +25 μg/mL CHL; +50 mM sodium propionate) in a 96 deep-well microtiter plate and grown to saturation overnight at 37 °C and 1050 rpm in a shaking incubator. The following day, cells were transferred into clear bottom, black microtiter plates, the OD_600_ was adjusted to 0.1–0.2 in 200 μL of LB (+100 μg/mL AMP; +25 μg/mL CHL and 50 mM sodium propionate), and cells continued to be grown at 37 °C and shaking at 1050 rpm. Following 3 to 5 h of incubation, microtiter plates were transferred into a TECAN Spark microtiter plate reader while monitoring the developing OD_600_ and the fluorescent signal (Ex. 480 ± 10 nm/Em. 525 ± 10 nm) at 30 °C. After approximately 45–90 min, either 4 μM rapamycin or an equivalent amount of DMSO was added, and the fluorescent signal was monitored for another 60 to 180 min.

## Supporting Information Available

Summary of library screens; results; and summary of amino acid sequences of iRapTors, LuciRapTors, FhuA variants, and linkers (PDF)
