# Supplementary material for: Dissecting the Permeability of the Escherichia coli Cell Envelope to a Small Molecule Using Tailored Intensiometric Fluorescent Protein Sensors
Source: ACS Omega. 2023 Oct 11;8(42):39562–9. doi: 10.1021/acsomega.3c05405 (PMC10601414; doi:10.1021/acsomega.3c05405)
Supplement: Supplementary file 1 — ao3c05405_si_001.pdf [file ao3c05405_si_001.pdf]

Supporting Information

Dissecting the permeability of the *Escherichia coli* cell envelope to a small molecule using tailored intensiometric fluorescent protein sensors

Authors

Philipp Kemp[1,2], Wadim Weber[1,2], Charlotte Desczyk[1], Marwan Kaufmann[1], Josefine Panthel[1], Theresa Wörmann[1], and Viktor Stein[1,2,*]

Affiliations

[1] Department of Biology, TU Darmstadt, 64287 Darmstadt, Germany;

[2] Centre for Synthetic Biology, TU Darmstadt, 64283 Darmstadt, Germany;

Corresponding Author

*Correspondence should be addressed to Viktor Stein; Tel. +49 6151 16 21947; Fax. +49 6151 16 22063; Email: stein@bio.tu-darmstadt.de

Supplementary Results

Library Screen #1 (Circular Permutated iRapTor)

Linker sequences of a select number of sensor variants following a sub-saturating screen of circular-permutated iRapTor variants are shown (**Tab. S1**). Gly residues imposed by the underlying iFLinkC DNA assembly process are generally included. The theoretical diversity of the underlying linker library comprised 400 iRapTor variants that were assembled from 20 defined co-polymeric linkers located in L1 and L2 (see **Tab. S4** for a summary of linker sequences and **Fig. 2A** for the topology of the circular-permutated iRapTor sensor). The ten best performing linker variants with a >1.9-fold switch-ON were then sequenced (**Tab. S1**).

Table S1. Summary of linker sequences of circular-permutated iRapTor variants

Variant Name	Linker		Occurrence
	L1	L2	
1-H10	GP_5G	GP_7G	1×
5-D2	GP_1G	GP_7G	1×
5-F12	GTPTPTPTG	GP_1G	1×
1-H8	GTPTPTPTG	GP_3G	1×
1-G3	GP_7G	GTPTG	1×
1-G1	GTPTG	GTPTPTPTG	1×
5-E2	GP_7G	GSPAG	1×
5-D6	GP_1G	GTPTPTPTG	1×
5-H5	GGSGGSGGSG	GP_3G	1×
5-F8	GTPTPTPTG	GP_1G	1×

Library Screen #2 (Domain-Inserted iRapTor)

Linker sequences of a select number of switch-ON and switch-OFF variants following a saturating library screen of 420 domain-inserted iRapTor variants are shown (**Tab. S2** and **Tab. S3**). Gly residues imposed by the underlying iFLinkC DNA assembly process are generally included. The theoretical diversity of the underlying library comprised 100 variants based on combinations of P_1 to P_{10} linkers in single Pro increments located in L1 and L3 (see **Fig. 2E** for the topology of domain-inserted iRapTor sensors). The library screen yielded 14 variants with >1.4-fold switch-ON and 5 variants with <0.7 switch-OFF that were further confirmed in cell lysates (see **Fig. 2G** and **2H** in the main manuscript for detailed measurements).

Table S2. Linker sequences of domain-inserted iRapTor >1.4-fold switch-ON

Variant Name	Linker Amino Acid Sequence			Occurrence
	L1	L2	L3	
P_4P_{10}	GP_4G	GP_7G	$GP_{10}G$	8×
P_3P_7	GP_3G	GP_7G	GP_7G	4×
P_4P_7	GP_4G	GP_7G	GP_7G	1×
P_1P_{10}	GP_1G	GP_7G	$GP_{10}G$	1×

Table S3. Linker sequences of domain-inserted iRapTor >0.7-fold switch-OFF

Variant Name	Linker			Occurrence
	L1	L2	L3	
P_1P_5	GPG	GP_7G	GP_5G	4×
P_1P_6	GPG	GP_7G	GP_6G	1×

Library Screen #3 (Domain-Inserted iRapTor without Gate Post Residues)

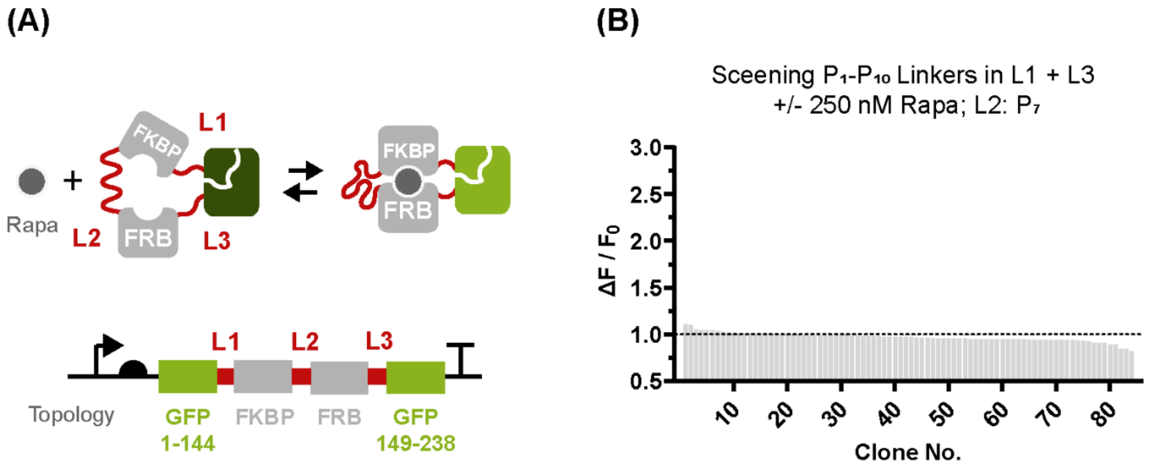

Fig. S1: Dissecting the contribution of gate post residues in the context of a domain-inserted iRapTor topology and minimal poly-Pro linkers. **(A)** Design principle: An allosteric receptor composed of FRB and FKBP12 was recombined with sfGFP, but this time inserted between position 144 and 149 with the so-called gate post residues removed. Insertion was achieved through combinations of minimal P_1 to P_{10} linkers featuring single Pro increments in L1 and L3. FRB and FKBP12 were generally separated by a P_7-linker in L2. The theoretical diversity of the underlying library comprised 100 poly-Pro linker variants of variable lengths; **(B)** Representative distribution in the response of individual iRapTor linker variants in the context of a domain-inserted topology. The library is largely unresponsive highlighting the need for gate post residues at position 145 and 149 in the context of minimal poly-Pro linkers.

Summary of Amino Acid Sequences of iRapTor Sensors and FhuA Nanopores

Affinity purification tags are highlighted in **bold**, NanoLuc in blue, sfGFP in green, FKBP12 in light grey and FRB in dark grey. The co-polymeric linker is denoted with XXX and flanked by two Gly residues required for compatibility with the underlying iFLinkC DNA assembly process

Amino Acid Sequences of iRapTor variants:

Circular-Permutated iRapTor^{H10TW} | for purification and in vitro characterisation

MGILWHEMWHEGLEEASRLYFGERNVKGMFEVLEPLHAMMERGPQTLKETSFNQAYGRDLMEAQEWCRKY
MKSGNVKDLTQAWDLYYHVFRRIGPPPPPGSHNVYITADKQKNGIKANFKIRHNVEDGSVQLADHYQQNT
PIGDGPVLLPDNHYLSTQSVLSKDPNEKRDHMVLLEFVTAAGITLGMDELYKGGTGGSMSKGEELFTGVV
PILVELDGDVNGHKFSVRGEGEGDATNGKLTLKFICTTGKLPVPWPTLVTTLTYGVQCFSRYPDHMKQHD
FFKSAMPEGYVQERTISFKDDGTYKTRAEVKFEGDTLVNRIELKGIDFKEDGNILGHKLEYNFNGPPPPP
PPGVQVETISPGDGRTFPKRGQTCVVHYTGMLEDGKKFDSSRDRNKPFKFMLGKQEVIRGWEEGVAQMSV
GQRAKLTISPDYAYGATGHPGIIPPHATLVFDVELLKLEAS**HHHHHH**

Circular-Permutated iRapTor^{H10TW} | for cellular assays

MGILWHEMWHEGLEEASRLYFGERNVKGMFEVLEPLHAMMERGPQTLKETSFNQAYGRDLMEAQEWCRKY
MKSGNVKDLTQAWDLYYHVFRRIGPPPPPGSHNVYITADKQKNGIKANFKIRHNVEDGSVQLADHYQQNT
PIGDGPVLLPDNHYLSTQSVLSKDPNEKRDHMVLLEFVTAAGITLGMDELYKGGTGGSMSKGEELFTGVV
PILVELDGDVNGHKFSVRGEGEGDATNGKLTLKFICTTGKLPVPWPTLVTTLTYGVQCFSRYPDHMKQHD
FFKSAMPEGYVQERTISFKDDGTYKTRAEVKFEGDTLVNRIELKGIDFKEDGNILGHKLEYNFNGPPPPP
PPGVQVETISPGDGRTFPKRGQTCVVHYTGMLEDGKKFDSSRDRNKPFKFMLGKQEVIRGWEEGVAQMSV
GQRAKLTISPDYAYGATGHPGIIPPHATLVFDVELLKLE

Circular-Permutated TorA-iRapTor^{H10TW} | for cellular assays

MGNNNDLFQASRRRLLAQLGNLTDAGTLGPSLLTPRRATAAQAGGSGILWHEMWHEGLEEASRLYFGERN
VKGMFEVLEPLHAMMERGPQTLKETSFNQAYGRDLMEAQEWCRKYMKSGNVKDLTQAWDLYYHVFRRIGP
PPPPGSHNVYITADKQKNGIKANFKIRHNVEDGSVQLADHYQQNTPIGDGPVLLPDNHYLSTQSVLSKDP
NEKRDHMVLLEFVTAAGITLGMDELYKGGTGGSMSKGEELFTGVVPILVELDGDVNGHKFSVRGEGEGDA
TNGKLTLKFICTTGKLPVPWPTLVTTLTYGVQCFSRYPDHMKQHDFFKSAMPEGYVQERTISFKDDGTYK
TRAEVKFEGDTLVNRIELKGIDFKEDGNILGHKLEYNFNGPPPPPPPGVQVETISPGDGRTFPKRGQTCV
VHYTGMLEDGKKFDSSRDRNKPFKFMLGKQEVIRGWEEGVAQMSVGQRAKLTISPDYAYGATGHPGIIPP

HATLVFDVELLKLE

Exemplary Domain-Inserted iRapTor | Including Gate Post Residues | Variant ***P_4P_{10}***

MGSKGEELFTGVVPILVELDGDVNGHKFSVRGEGEGDATNGKLTLKFICTTGKLPVPWPTLVTTLTYGVQCFSRYPDHMKRHDFFKSAMPEGYVQERTISFKDDGTYKTRAEVKFEGDTLVNRIELKGIDFKEDGNILGHKLEYNFGPPPPGVQVETISPGDGRTFPKRGQTCVVHYTGMLEDGKKFDSSRDRNKPFKFMLGKQEVIRGWEEGVAQMSVGQRAKLTISPDYAYGATGHPGIIPPHATLVFDVELLKLEGPPPPPPPGILWHEMWHEGLEEASRLYFGERNVKGMFEVLEPLHAMMERGPQTLKETSFNQAYGRDLMEAQEWCRKYMKSGNVKDLTQAWDLYYHVFRRIGPPPPPPPPPPGHNVYITADKQKNGIKANFKIRHNVEDGSVQLADHYQQNTPIGDGPVLLPDNHYLSTQSVLSKDPNEKRDHMVLLEFVTAAGITHGMDELYK**HHHHHH**

Domain-Inserted iRapTor Library | Excluding Gate Post Residues | See ***Fig. S3*** *for screen*

MGSKGEELFTGVVPILVELDGDVNGHKFSVRGEGEGDATNGKLTLKFICTTGKLPVPWPTLVTTLTYGVQCFSRYPDHMKRHDFFKSAMPEGYVQERTISFKDDGTYKTRAEVKFEGDTLVNRIELKGIDFKEDGNILGHKLEYNGP_1//P_{10}GVQVETISPGDGRTFPKRGQTCVVHYTGMLEDGKKFDSSRDRNKPFKFMLGKQEVIRGWEEGVAQMSVGQRAKLTISPDYAYGATGHPGIIPPHATLVFDVELLKLEGPPPPPPPGILWHEMWHEGLEEASRLYFGERNVKGMFEVLEPLHAMMERGPQTLKETSFNQAYGRDLMEAQEWCRKYMKSGNVKDLTQAWDLYYHVFRRIGP_1//P_{10}GNVYITADKQKNGIKANFKIRHNVEDGSVQLADHYQQNTPIGDGPVLLPDNHYLSTQSVLSKDPNEKRDHMVLLEFVTAAGITHGMDELYK**HHHHHH**

<u>Amino Acid Sequences of bioluminescent LuciRapTor variants:</u>

Circular-Permutated LuciRapTor^{H10TW} | Variant ***3B02***

MGILWHEMWHEGLEEASRLYFGERNVKGMFEVLEPLHAMMERGPQTLKETSFNQAYGRDLMEAQEWCRKYMKSGNVKDLTQAWDLYYHVFRRIGPPPPPGSHNVYITADKQKNGIKANFKIRHNVEDGSVQLADHYQQNTPIGDGPVLLPDNHYLSTQSVLSKDPNEKRDHMVLLEFVTAAGITLGMDELYKGPPPPPPPPPPGVFTLEDFVGDWRQTAGYNLDQVLEQGGVSSLFQNLGVSVTPIQRIVLSGENGLKIDIHVIIPYEGLSGDQMGQIEKIFKVVYPVDDHHFKVILHYGTLVIDGVTPNMIDYFGRPYEGIAVFDGKKITVTGTLWNGNKIIDERLINPDGSLLFRVTINGVTGWRLCERGPPPGMSKGEELFTGVVPILVELDGDVNGHKFSVRGEGEGDATNGKLTLKFICTTGKLPVPWPTLVTTLTYGVQCFSRYPDHMKQHDFFKSAMPEGYVQERTISFKDDGTYKTRAEVKFEGDTLVNRIELKGIDFKEDGNILGHKLEYNFNGPPPPPPPGVQVETISPGDGRTFPKRGQTCVVHYTGMLEDGKKFDSSRDRNKPFKFMLGKQEVIRGWEEGVAQMSVGQRAKLTISPDYAYGATGHPGIIPPHATLVFDVELLKLEAS**HHHHHH**

*Exemplary Domain-Inserted LuciRapTor | Including Gate Post Residues | Variant **3H8***

MGVFTLEDFVGDWRQTAGYNLDQVLEQGGVSSLFQNLGVSVTPIQRIVLSGENGLKIDIHVIIPYEGLSG
DQMGQIEKIFKVVYPVDDHHFKVILHYGTLVIDGVTPNMIDYFGRPYEGIAVFDGKKITVTGTLWNGNKI
IDERLINPDGSLLFRVTINGVTGWRLCERGSKGEELFTGVVPILVELDGDVNGHKFSVRGEGEGDATNGK
LTLKFICTTGKLPVPWPTLVTTLTYGVQCFSRYPDHMKRHDFFKSAMPEGYVQERTISFKDDGTYKTRAE
VKFEGDTLVNRIELKGIDFKEDGNILGHKLEYNFGPPPPGVQVETISPGDGRTFPKRGQTCVVHYTGMLE
DGKKFDSSRDRNKPFKFMLGKQEVIRGWEEGVAQMSVGQRAKLTISPDYAYGATGHPGIIPPHATLVFDV
ELLKLEGPPPPPPPGILWHEMWHEGLEEASRLYFGERNVKGMFEVLEPLHAMMERGPQTLKETSFNQAYG
RDLMEAQEWCRKYMKSGNVKDLTQAWDLYYHVFRRIGPPPPPPPGHNVYITADKQKNGIKANFKIRHNVE
DGSVQLADHYQQNTPIGDGPVLLPDNHYLSTQSVLSKDPNEKRDHMVLLEFVTAAGITHGMDELYK**HHHH
HH**

Amino Acid Sequences of FhuA variants:

Signal peptide in orange, FhuA variants in gold

FhuA^{WT}

MGARSKTAQPKHSLRKIAVVVATAVSGMSVYAQAAVEPKEDTITVTAAPAPQESAWGPAATIAARQSATG
TKTDTPIQKVPQSISVVTAEEMALHQPKSVKEALSYTPGVSVGTRGASNTYDHLIIRGFAAEGQSQNNYL
NGLKLQGNFYNDAVIDPYMLERAEIMRGPVSVLYGKSSPGGLLNMVSKRPTTEPLKEVQFKAGTDSLFQT
GFDFSDSLDDDGVYSYRLTGLARSANAQQKGSEEQRYAIAPAFTWRPDDKTNFTFLSYFQNEPETGYYGW
LPKEGTVEPLPNGKRLPTDFNEGAKNNTYSRNEKMVGYSFDHEFNDTFTVRQNLRFAENKTSQNSVYGYG
VCSDPANAYSKQCAALAPADKGHYLARKYVVDDEKLQNFSVDTQLQSKFATGDIDHTLLTGVDFMRMRND
INAWFGYDDSVPLLNLYNPVNTDFDFNAKDPANSGPYRILNKQKQTGVYVQDQAQWDKVLVTLGGRYDWA
DQESLNRVAGTTDKRDDKQFTWRGGVNYLFDNGVTPYFSYSESFEPSSQVGKDGNIFAPSKGKQYEVGVK
YVPEDRPIVVTGAVYNLTKTNNLMADPEGSFFSVEGGEIRARGVEIEAKAALSASVNVVGSYTYTDAEYT
TDTTYKGNTPAQVPKHMASLWADYTFFDGPLSGLTLGTGGRYTGSSYGDPANSFKVGSYTVVDALVRYDL
ARVGMAGSNVALHVNNLFDREYVASCFNTYGCFWGAERQVVATATFRF

Cork-less FhuA$^{\Delta C \Delta 5L}$

MGARSKTAQPKHSLRKIAVVVATAVSGMSVYAQAGLKEVQFKAGTDSLFQTGFDFSDSLDDDGVYSYRLT
GLARSANAQQKGSEEQRYAIAPAFTWRPDDKTNFTFLSYFQNEPETGNSEGSTYSRNEKMVGYSFDHEFN
DTFTVRQNLRFAENKTSQNSVYGNSEGSRKYVVDDEKLQNFSVDTQLQSKFATGDIDHTLLTGVDFMRMR
NDINAWFGYNSEGSSGPYRILNKQKQTGVYVQDQAQWDKVLVTLGGRYDWADQESLNRVAGTTDKRDDKQ
FTWRGGVNYLFDNGVTPYFSYSESFEPSSQVGKDGNIFAPSKGKQYEVGVKYVPEDRPIVVTGAVYNLTK
TNNLMADPEGSFFSVEGGEIRARGVEIEAKAALSASVNVVGSYTYTDAEYTTDTTYKGNTPAQVPKHMAS
LWADYTFFDGPLSGLTLGTGGRYTNSEGSYTVVDALVRYDLARVGMAGSNVALHVNSEGSQVVATATFRF

Table S4. Linker sequences used in the construction of circular-permutated iRapTor

Denomination	Amino Acid Sequence	Length
P3	GPG	3
P5	GPPPG	5
P7	GPPPPPG	7
P8	GPPPPPPG	8
P9	GPPPPPPPG	9
TP5	GTPTG	5
TP7	GTPTPTG	7
TP9	GTPTPTPTG	9
TP11	GTPTPTPTPTPTG	11
EAAAK16	GGAEAAAKEAAAKAGG	16
EAAAK29	GGAEAAAKAGSAEAAAKAGSAEAAAKAGG	29
PAS5	GSPAG	5
PAS8	GGASPAGG	8
G1	G	1
G2	GG	2
G3	GGG	3
GS4	GGSG	4
GS7	GGSGGSG	7
GS10	GGSGGSGGSG	10
GS15	GGSGGSGGSGGSGSG	15

Table S5. Linker sequences used to construct domain-inserted and bioluminescent iRapTor

Denomination	Amino Acid Sequence	Length
P3	GPG	3
P4	GPPG	4
P5	GPPPG	5
P6	GPPPPG	6
P7	GPPPPPG	7
P8	GPPPPPPG	8
P9	GPPPPPPPG	9
P10	GPPPPPPPPG	10
P11	GPPPPPPPPPG	11
P12	GPPPPPPPPPPG	12
